# Using arterial-venous oxygen difference to guide red blood cell transfusion strategy

**DOI:** 10.1186/s13054-020-2827-5

**Published:** 2020-04-20

**Authors:** Alberto Fogagnolo, Fabio Silvio Taccone, Jean Louis Vincent, Giulia Benetto, Elaine Cavalcante, Elisabetta Marangoni, Riccardo Ragazzi, Jacques Creteur, Carlo Alberto Volta, Savino Spadaro

**Affiliations:** 1 Department of Morphology, Surgery and Experimental Medicine, Section of Anaesthesia and Intensive Care, Azienda Ospedaliera-Universitaria Sant’ Anna, University of Ferrara, 8, Aldo Moro, 44121 Ferrara, Italy; 2 Department of Intensive Care, Erasme Hospital, Université Libre de Bruxelles, Brussels, Belgium

**Keywords:** Red blood cell transfusion, Oxygen consumption, Critical care, Mortality

## Abstract

**Background:**

Guidelines recommend a restrictive red blood cell transfusion strategy based on hemoglobin (Hb) concentrations in critically ill patients. We hypothesized that the arterial-venous oxygen difference (A-V O_2diff_), a surrogate for the oxygen delivery to consumption ratio, could provide a more personalized approach to identify patients who may benefit from transfusion.

**Methods:**

A prospective observational study including 177 non-bleeding adult patients with a Hb concentration of 7.0–10.0 g/dL within 72 h after ICU admission. The A-V O_2diff_, central venous oxygen saturation (ScvO_2_), and oxygen extraction ratio (O_2_ER) were noted when a patient’s Hb was first within this range. Transfusion decisions were made by the treating physician according to institutional policy. We used the median A-V O_2diff_ value in the study cohort (3.7 mL) to classify the transfusion strategy in each patient as “appropriate” (patient transfused when the A-V O_2diff_ > 3.7 mL or not transfused when the A-V O_2diff_ ≤ 3.7 mL) or “inappropriate” (patient transfused when the A-V O_2diff_ ≤ 3.7 mL or not transfused when the A-V O_2diff_ > 3.7 mL). The primary outcome was 90-day mortality.

**Results:**

Patients managed with an “appropriate” strategy had lower mortality rates (23/96 [24%] vs. 36/81 [44%]; *p* = 0.004), and an “appropriate” strategy was independently associated with reduced mortality (hazard ratio [HR] 0.51 [95% CI 0.30–0.89], *p* = 0.01). There was a trend to less acute kidney injury with the “appropriate” than with the “inappropriate” strategy (13% vs. 26%, *p* = 0.06), and the Sequential Organ Failure Assessment (SOFA) score decreased more rapidly (*p* = 0.01). The A-V O_2diff_, but not the ScvO_2_, predicted 90-day mortality in transfused (AUROC = 0.656) and non-transfused (AUROC = 0.630) patients with moderate accuracy. Using the ROC curve analysis, the best A-V O_2diff_ cutoffs for predicting mortality were 3.6 mL in transfused and 3.5 mL in non-transfused patients.

**Conclusions:**

In anemic, non-bleeding critically ill patients, transfusion may be associated with lower 90-day mortality and morbidity in patients with higher A-V O_2diff_.

**Trial registration:**

ClinicalTrials.gov, NCT03767127. Retrospectively registered on 6 December 2018.

## Background

The decision whether or not to transfuse an anemic patient is a considerable challenge in the intensive care unit (ICU) and should always consider the balance between the risks of anemia and the potential harms of red blood cell (RBC) transfusion in that patient. In critically ill patients, global oxygen consumption (VO_2_) is often increased, so that anemia can lead to impaired tissue oxygenation and contribute to adverse outcomes, including failed weaning from mechanical ventilation [1], acute kidney injury (AKI) [2], and increased mortality [3, 4]. However, transfusions are also associated with well-described risks, including transfusion-transmitted infections, transfusion-related acute lung injury (TRALI), transfusion-associated circulatory overload (TACO), and acquired immunosuppression [5, 6].

More than one fourth of critically ill patients receive a transfusion during their ICU stay [7], with the primary aim to increase global oxygen delivery (DO_2_) and decrease the DO_2_ to VO_2_ ratio [8, 9]. Clinical guidelines recommend a restrictive RBC transfusion strategy, i.e., transfusions limited to patients with a hemoglobin (Hb) concentration < 7 g/dL [10]. Of note, the guideline recommendations only refer to clinically stable intensive care patients, and there is still equipoise around the optimal transfusion strategy in several subgroups of patients. For example, a higher threshold of 8 or even 9 g/dL may be considered in patients with pre-existing cardiovascular disease [10]. The guidelines are based on results from several randomized clinical studies that showed that liberal transfusion strategies (keeping Hb > 9–10 g/dL) provided no clinical benefit in different populations when compared to a restrictive strategy [11–14]. However, the decision to transfuse should not be based only on Hb concentration [15, 16], because the optimal DO_2_ can differ from one patient to another [17] and over time in any one patient. These elements have resulted in considerable variability in worldwide transfusion practice, with the average Hb concentrations used to initiate transfusions higher than those recommended [7].

It has been suggested that mixed venous oxygen saturation (SvO_2_), which reflects the balance between VO_2_ and DO_2_, could be a valuable guide for transfusion in critically ill patients [18]. We hypothesized that patients with a greater arterial-venous oxygen difference (A-V O_2diff_), a surrogate for the ratio between DO_2_ and VO_2_, may benefit more from RBC transfusion than patients with lower A-V O_2diff_, who may benefit from avoiding transfusion. The aim of this observational study was therefore to evaluate whether “appropriate” RBC transfusions, as defined using the A-V O_2diff_, may be associated with reduced mortality rates and severity of organ dysfunction in non-acutely bleeding critically ill patients.

## Methods

### Study population

This prospective observational study was conducted in the Department of Intensive Care at the University Hospital of Ferrara (Italy) from 1 January 2017 to 1 October 2018. The study was approved by the local ethics committee (protocol number 160699, date of approval 14 July 2016). We included non-bleeding adult patients who had a Hb concentration between 7.0 and 10.0 g/dL within 72 h after admission to the ICU. All patients with a Hb concentration within the target range (7.0–10.0 g/dL) were included, regardless of whether or not a transfusion was given. Exclusion criteria were as follows: age < 18 years, acute bleeding, pregnancy, and no central venous catheter or central venous catheter placed in a femoral vein. Patients who were readmitted during the study period were not included a second time. Written informed consent was given by the patient or their next of kin. The manuscript is reported according to the STROBE criteria [19].

### Transfusion policy

The decision to transfuse was made by the physician in charge of the patient according to our local transfusion policy, which includes [20–22] the following:

- Hb target of 7–10 g/dL
- A clinical decision that transfusion is appropriate, in addition to the Hb concentration
- A single-unit transfusion strategy
- A “first-in, first-out” policy (the oldest units are used first), to minimize blood wastage

RBC packs transfused during the study period were stored in saline, adenine, glucose, mannitol (SAGM) additive solution for up to 42 days at a mean temperature of 4 ± 2 °C. The RBC units were all leuko-reduced, according to the policy of our hospital during the study period. Characteristics of the RBC units used (i.e., days of storage, characteristics of donor) were not recorded.

### Definitions

Chronic anemia was defined based on a known history of anemia and/or a decreased hematocrit (Hct) and Hb concentration on previous laboratory tests [23]. AKI was defined according to the Kidney Disease: Improving Global Outcomes (KDIGO) guidelines as an increase in serum creatinine of 0.3 mg/dL within 48 h, an increase in serum creatinine to 1.5 times the baseline value present within the previous 7 days, or urine volume < 0.5 mL/kg/h for 6 h [24]. Chronic renal disease was defined according to the same KDIGO guidelines [24].

### Data collection

The Simplified Acute Physiology Score (SAPS) was recorded on admission. Demographic data and clinical variables, including heart rate, arterial pressure Glasgow coma scale score, and the Richmond Agitation-Sedation Scale, were recorded at the time of study inclusion (i.e., the first point at which the Hb concentration was measured as being between 7.0 and 10.0 g/dL). The Sequential Organ Failure Assessment (SOFA) score was calculated at the time of study inclusion and on the subsequent 5 days. Results of routine arterial and central venous blood sampling for blood gas analysis (GEM Premier™ 4000, Werfen, Le Pré-Saint-Gervais, France) and hematologic variables (Sysmex K-1000 auto-analyzer, Block Scientific, Bohemia, NY, USA) were recorded at the time of study inclusion. Decisions to transfuse on the day of study inclusion were also noted. Hb concentrations were measured at least daily as part of the routine practice and recorded 5 days after study inclusion. The number of RBC units given was also recorded for the first 5 days after study inclusion.

The A-V O_2diff_ was calculated on the day of study inclusion as the difference between arterial oxygen content (CaO_2_) and central venous oxygen content (CcvO_2_) where:

$$ {\mathrm{CaO}}_2={\mathrm{SaO}}_2\times \mathrm{Hb}\times 1.39+\left({\mathrm{PaO}}_2\times 0.031\right) $$

and

$$ {\mathrm{CcvO}}_2={\mathrm{ScvO}}_2\times \mathrm{Hb}\times 1.39+\left({\mathrm{PcvO}}_2\times 0.031\right). $$

We also calculated the oxygen extraction ratio (O_2_ER), defined as [(CaO_2_ − CcvO_2_)/CaO_2_]. Using the median A-V O_2diff_ of our patients as the cutoff, we retrospectively categorized patients as having high A-V O_2diff_ (greater than the median value) or low A-V O_2diff_ (less than or equal to the median value). We then grouped patients into those who had been managed using an “appropriate” (transfused if A-V O_2diff_ was high, not transfused if A-V O_2diff_ was low) or an “inappropriate” (transfused if A-V O_2diff_ was low, not transfused if A-V O_2diff_ was high) transfusion strategy (Supplemental Figure S1). A patient was considered “non-transfused” if he/she did not receive RBC units on the day of study inclusion.

Treating physicians were not blinded to the A-V O_2diff_ results, but were not supposed to use them to influence decisions to transfuse.

### Outcomes

The primary outcome of the study was 90-day mortality. Secondary outcomes were the occurrence of AKI, the ICU length of stay, the mechanical ventilation- and vasopressor-free days over the 28 days post-study inclusion, the time course of organ dysfunction as assessed by the SOFA score over the 5 days post-study inclusion (the worst daily value was recorded for analysis), and the daily Hb concentrations.

### Sample size

The sample size was calculated from a pilot study, presented as a congress abstract [25], in which the ratio of “appropriate” to “inappropriate” transfusion strategy was 1.38, with a mortality of 35% in the “inappropriate” group and 17% in the “appropriate” group. We therefore calculated that 148 patients should be enrolled to have 80% power to detect an 18% difference in 90-day mortality between the groups with an alfa error of 5%. Taking into account up to 20% loss to follow-up and a variation in the “appropriate”/“inappropriate” transfusion ratio, we planned to enroll at least 175 patients.

### Statistical analysis

Normal distribution of data was tested using the Shapiro–Wilk normality test. Data are reported as mean ± standard deviation or median [interquartile range] when appropriate. Unpaired Student’s *t* tests or Mann–Whitney *U* tests were used for data with normal and non-normal distributions, respectively. ROC curves were used to analyze the ability of A-V O_2diff_ to predict 90-day mortality in each group. Optimal cutoff scores for A-V O_2diff_ were determined using Youden’s index. Differences in repeated measurements in the two groups were analyzed using repeated measure ANOVA or Friedman’s rank analysis for normally and not normally distributed variables, respectively. When multiple comparisons were made, *p* values were adjusted using the Bonferroni post hoc procedure. Mortality was also analyzed by quartiles of A-V O_2diff_ in the “appropriate” and “inappropriate” groups. Multivariable logistic regression models were performed to investigate predictors of mortality; we included as covariates in the model all variables with *p* ≤ 0.10 at univariate analysis. Finally, a Cox proportional hazard regression model was used to obtain hazard ratios (HRs) for 90-day mortality. The analysis of ICU length of stay was only performed on patients discharged alive.

Four post hoc analyses were also performed. The first was to describe the time course of non-renal SOFA score over the first 5 days after study inclusion. The second was to identify the A-V O_2diff_ cutoffs that were the best predictors of mortality in the whole population and in transfused and non-transfused patients. The third was to compare the results obtained using the A-V O_2diff_ as a physiological marker of an “appropriate” strategy with those obtained using ScvO_2_ and O_2_ER as other oxygen-derived parameters. For this purpose, the area under the A-V O_2diff_ receiver operating characteristic (AUROC) curve for 90-day mortality was compared with the AUROCs for ScvO_2_ and O_2_ER, using the median ScvO_2_ and O_2_ER values to define ScvO_2_-based and O_2_ER-based “appropriate” and “inappropriate” strategies. Finally, for patients who had a Hb concentration ≤ 10 g/dL at any time during the first 5 days after study inclusion, we calculated whether the transfusion strategy was “appropriate” or not on each day. We compared the rates of “appropriate” transfusion strategy in survivors and non-survivors, and mortality rates in patients in whom all transfusions were “appropriate” and all were “inappropriate.”

A *p* value < 0.05 was considered statistically significant. Statistical analyses were performed using SPSS Statistics for Windows, version 25.0 (IBM, Armonk, NY, USA).

## Results

### Study population

During the study period, 212 patients were screened for eligibility; 177 met the inclusion criteria and were enrolled (Supplemental Figure S2). The most common reasons for ICU admission were sepsis/septic shock (*n* = 58 [33%]) and respiratory failure (*n* = 50 [28%]) (Table 1).

**Table 1:** Clinical and demographic characteristics

Characteristic	All patients (*n* = 177)	“Appropriate” strategy (*n* = 96)	“Inappropriate” strategy (*n* = 81)	*p* value
Age, years	72 ± 12	71 ± 14	72 ± 10	0.73
BMI, kg/m^2^	28 ± 5	27 ± 4	29 ± 6	0.08
SAPS II score at admission	42 [29–50]	42 [30–48]	45 [33–55]	0.06
SOFA score at admission	5 [2–6]	5 [3–6]	5 [2–6]	0.81
SOFA score at study inclusion	5 [3–7]	5 [3–7]	5 [3–7]	0.46
RASS score at study inclusion	− 1 [− 2; 1]	− 2 [− 2; 1]	− 1 [− 2; 1]	0.33
ICU days before study inclusion	1 [0–2]	1 [0–2]	1 [0–2]	0.91
Transfused, *n* (%)	95 (54)	48 (50)	47 (58)	0.36
Comorbidity				
Heart disease, *n* (%)	102 (58)	58 (60)	44 (54)	0.50
Hypertension, *n* (%)	118 (67)	62 (65)	56 (69)	0.63
Diabetes, *n* (%)	53 (30)	26 (27)	27 (33)	0.46
Chronic anemia, *n* (%)	28 (16)	15 (16)	13 (16)	0.99
COPD/asthma, *n* (%)	30 (17)	11 (11)	19 (23)	0.06
History of smoking, *n* (%)	45 (26)	25 (26)	14 (17)	0.22
Chronic renal disease, *n* (%)	49 (28)	22 (23)	27 (33)	0.22
Reason for admission				
Sepsis/septic shock, *n* (%)	58 (33)	30 (31)	28 (35)	0.76
Respiratory failure, *n* (%)	50 (28)	30 (31)	20 (25)	0.42
Hypovolemic shock, *n* (%)	28 (16)	15 (16)	13 (16)	0.94
Cardiogenic shock, *n* (%)	19 (11)	6 (6)	13 (16)	0.06
Trauma, *n* (%)	8 (4)	4 (4)	4 (5)	0.80
Others	13 (7)	11 (11)	3 (1)	0.10
Interventions on admission				
Mechanical ventilation, *n* (%)	157 (88)	88 (93)	69 (85)	0.18
Vasopressors, *n* (%)	81 (46)	47 (49)	34 (42)	0.40
Laboratory values on inclusion				
Hemoglobin, g/dL	8.7 ± 0.7	8.8 ± 0.6	8.6 ± 0.8	0.06
MCV, fL	88 ± 9	90 ± 6	87 ± 11	0.07
RDW, %	15.9 ± 3.0	15.4 ± 2.5	16.4 ± 3.3	0.02
Platelets, 10^3^/μL	179 [129–266]	178 [132–282]	189 [126–244]	0.72
INR	1.27 ± 0.3	1.26 ± 0.3	1.32 ± 0.3	0.12
Creatinine, mg/dL	1.09 [0.89–2.01]	1.08 [0.77–1.96]	1.10 [0.98–2.40]	0.15
Bilirubin, mg/dL	0.72 [0.41–1.10]	0.85 [0.45–1.00]	0.63 [0.41–1.00]	0.26
Lactate, mmol/L	1.6 [1.1–2.0]	1.4 [1.0–2.0]	2.0 [1.2–2.0]	0.04
CaO_2_, mL	12.4 ± 1.6	12.5 ± 1.1	12.2 ± 2.1	0.26
ScvO_2_, %	71 ± 9	71 ± 10	73 ± 9	0.16

*BMI* body mass index, *SAPS* Simplified Acute Physiology Score, *RASS* Richmond Agitation-Sedation Scale, *COPD* chronic obstructive pulmonary disease, *RDW* red blood cell distribution width, *MCV* mean corpuscular volume, *INR* international normalized ratio, *CaO*_*2*_ arterial oxygen content, *ScvO*_*2*_ central venous oxygen saturation

The median A-V O_2diff_ of the whole population was 3.7 mL, and using this value, 96 patients (54%) were considered to have been managed using an “appropriate” transfusion strategy and 81 (46%) using an “inappropriate” strategy. The transfusion rates in the “appropriate” and “inappropriate” groups were 50% (48/96) and 58% (47/81), respectively (*p* = 0.36). The mean lowest Hb concentration before transfusion was 8.3 ± 0.7 (range 7.0–9.8) g/dL, and the mean first Hb after transfusion was 9.0 ± 0.7 g/dL (range 7.7–11.3). There were no significant differences in Hb concentrations between the groups during the study period (Supplemental Figure S3). The CaO_2_ at study inclusion and the proportion of patients with a history of chronic anemia did not differ between the groups (Table 1).

### Ninety-day mortality

**Fig. 1:**
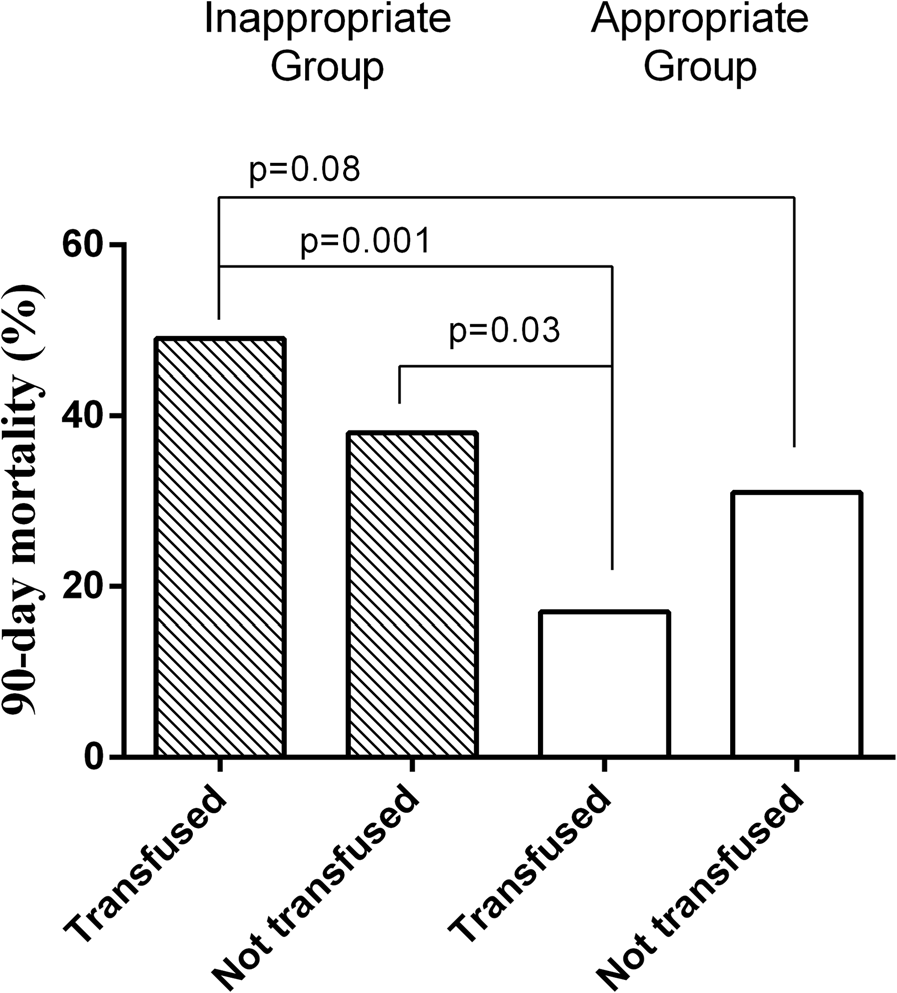
Ninety-day mortality in transfused and non-transfused patients in each group

The 90-day mortality was 33% (59/177) and was similar in transfused and non-transfused patients (31/95 [33%] vs. 28/82 [34%]; *p* = 0.87). The RBC transfusion rate was not significantly different in survivors and non-survivors (Supplemental Table 1). Patients in the “appropriate” strategy group had a lower mortality rate than the other patients (23/96 [24%] vs. 36/81 [44%]; odds ratio [OR] = 0.39 [95% CI 0.21–0.75], *p* = 0.004); this was mainly evident in the patients who received a transfusion (8/48 [17%] vs. 23/47 [49%]; *p* = 0.001) (Fig. 1). The association between “appropriate” strategy and lower mortality rate was confirmed in a subgroup analysis taking into account different Hb levels (Online Supplement).

**Fig. 2:**
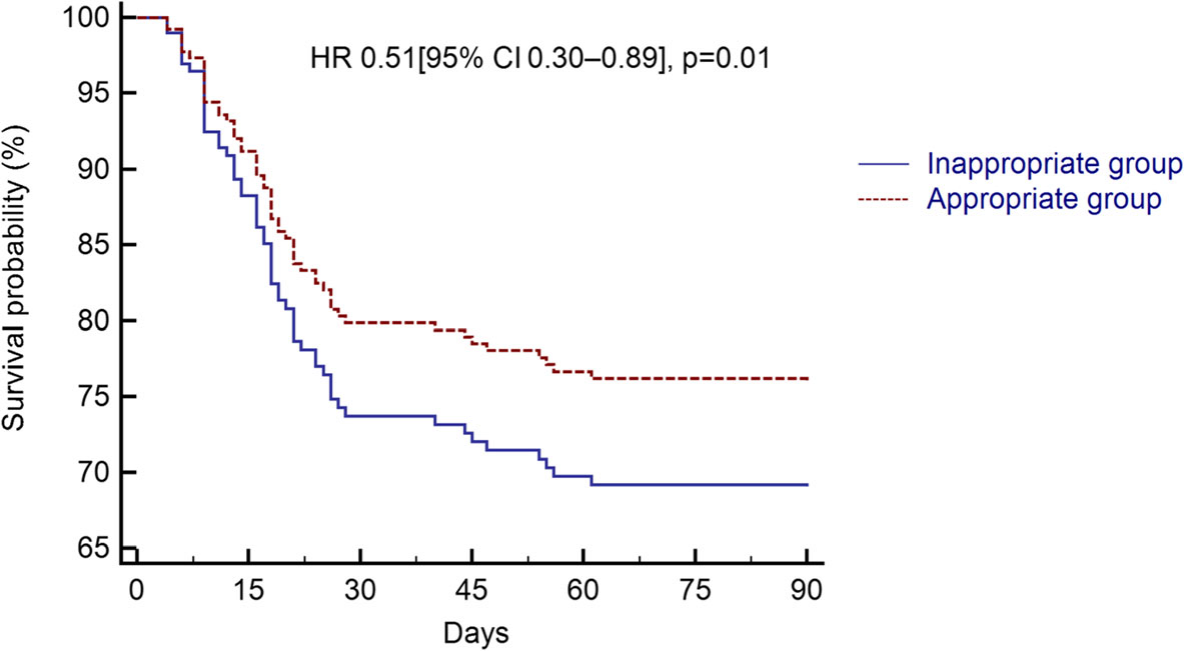
Cox regression analysis for 90-day mortality in the “appropriate” and “inappropriate” groups

Higher SAPS II score, RBC distribution width (RDW) values, and lactate concentrations on admission were associated with higher 90-day mortality in univariate analysis, but these were not significant after multivariable adjustment (Table 2). The use of an “appropriate” transfusion strategy was independently associated with lower 90-day mortality in a multivariable-adjusted Cox regression (HR 0.51 [95% CI 0.30–0.89], *p* = 0.01) (Table 2, Fig. 2). A-V O_2diff_ as a continuous variable was associated with 90-day mortality in non-transfused patients (OR = 1.50 [95% CI 1.02–2.20], *p* = 0.04); this association was confirmed in the multivariable analysis (Supplemental Table 2).

**Table 2:** Univariate and multivariable analyses with 90-day mortality as the dependent variable

Variables	Unadjusted odds ratio	*p* value	Adjusted odds ratio	*p* value
Age, years	1.02 [0.98–1.03]	0.85		
BMI, kg/m^2^	1.09 [0.99–1.20]	0.73		
SAPS II	1.02 [1.00–1.05]	0.05	1.01 [0.99–1.04]	0.39
Transfused, *n* (%)	0.93 [0.49–1.74]	0.83		
“Appropriate” group	0.39 [0.21–0.75]	0.004	0.48 [0.25–0.92]	0.03
SOFA score	1.07 [0.95–1.22]	0.26		
Comorbidity				
Heart disease, *n* (%)	1.17 [0.56–2.42]	0.57		
Hypertension, *n* (%)	1.18 [0.55–2.55]	0.67		
Diabetes, *n* (%)	1.74 [0.81–3.75]	0.16		
COPD/asthma, *n* (%)	0.61 [0.23–1.65]	0.33		
History of smoking, *n* (%)	1.02 [0.57–2.52]	0.98		
Chronic renal disease, *n* (%)	1.10 [0.49–2.44]	0.81		
Laboratory values on inclusion				
Hemoglobin, g/dL	1.16 [0.74–1.81]	0.51		
Platelets, 10^3^/μL	0.99 [0.99–1.01]	0.20		
INR	1.57 [0.42–5.79]	0.49		
RDW, %	1.12 [1.01–1.25]	0.04	1.11 [0.99–1.24]	0.07
Creatinine, mg/dL	1.03 [0.92–1.29]	0.68		
Bilirubin, mg/dL	1.35 [0.84–2.18]	0.21		
Lactate, mmol/L	1.36 [1.04–1.78]	0.02	1.22 [0.95–1.59]	0.12
PaO_2_/F_i_O_2_ ratio	0.99 [0.99–1.13]	0.62		

*BMI* body mass index, *SAPS* Simplified Acute Physiology Score, *COPD* chronic obstructive pulmonary disease, *RDW* red blood cell distribution width, *INR* international normalized ratio, *PaO*_*2*_ partial pressure of oxygen, *F*_*i*_*O*_*2*_ fraction of inspired oxygen

**Fig. 3:**
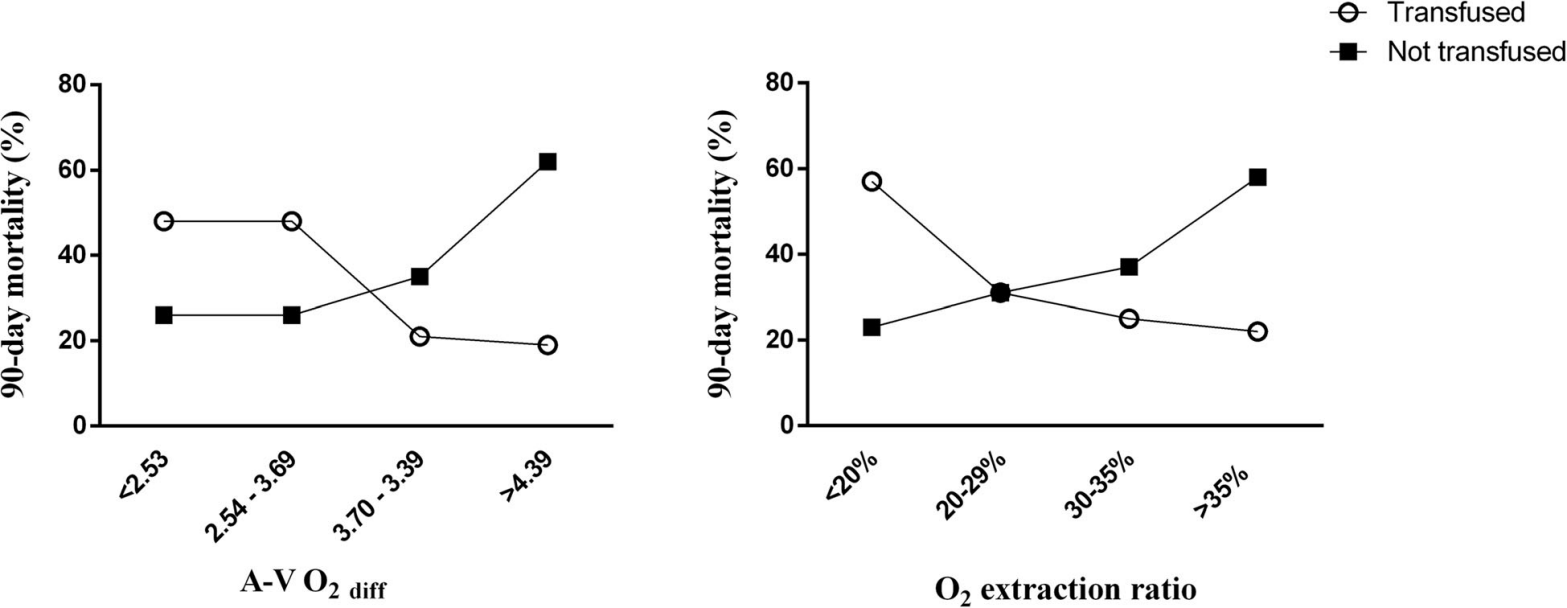
Ninety-day mortality in transfused and non-transfused patients according to quartiles of arterial-venous oxygen difference (A-V O_2diff_) and O_2_ extraction ratio

In all non-transfused patients, mortality increased across increasing A-V O_2diff_ quartiles; in all transfused patients, mortality decreased over A-V O_2diff_ quartiles (Fig. 3). The ROC analysis showed that the A-V O_2diff_ was a moderate independent predictor of 90-day mortality in transfused (AUROC = 0.656, best cutoff = 3.6 mL) and non-transfused (AUROC = 0.630, best cutoff = 3.5 mL) patients.

### Secondary outcomes and post hoc analyses

Fewer patients in the “appropriate” than in the “inappropriate” strategy group developed AKI (21/81 [26%] vs. 13/96 [13%]; *p* = 0.06) (Supplemental Table 3). The number of patients transfused during the first 5 days after study inclusion was similar in the two groups as was the median number of RBC units given (Supplemental Table 3). The SOFA score decreased more rapidly in patients managed with the “appropriate” strategy than in other patients (Supplemental Figure S4; *p* = 0.019). To avoid cross-interactions between the occurrence of AKI and the SOFA score evaluation, we also analyzed the non-renal SOFA scores, which gave similar results (*p* = 0.009 for comparison between “appropriate” and “inappropriate” strategies; data not shown).

Mortality prediction was better for A-V O_2diff_ than for ScvO_2_ (AUROC = 0.489 in transfused and AUROC = 0.440 in non-transfused) and O_2_ER (AUROC = 0.623 in transfused and AUROC = 0.619 in non-transfused). An O_2_ER-based “appropriate” strategy (using the median value of 29%) was also independently associated with a lower 90-day mortality (OR 0.44 [95% CI 0.23–0.86]; *p* = 0.02) (Supplemental Table 4), but a ScvO_2_-based “appropriate” strategy (using the median value of 71.5%) was not (OR 0.60 [95% CI 0.32–1.13]; *p* = 0.11).

When we divided the population using the best A-V O_2diff_ cutoffs for predicting mortality determined by ROC curve analysis (i.e., 3.6 mL in transfused and 3.5 mL in non-transfused patients) rather than the median value, we identified 94 patients who were “appropriately” transfused. There was a significant association between 90-day mortality and “appropriateness” of transfusion even when these cutoffs were used (HR 0.46 [95% CI 0.27–0.77]; *p* = 0.004).

During the first 5 days after study inclusion, 100 patients had a Hb concentration ≤ 10 g/dL on day 2, 97 patients on day 3, 64 on day 4, and 64 on day 5. During the study period, the transfusion strategy when the Hb was ≤ 10 g/dL was always “appropriate” in 76 of the patients and always “inappropriate” in 50 patients; the mortality rates were 20% (15/76) and 56% (28/50), respectively (*p* < 0.001).

## Discussion

The main result of this study is that the evaluation of the A-V O_2diff_ can identify critically ill patients who seem to benefit from RBC transfusion. Indeed, patients included in the “appropriate” transfusion group, defined according to the A-V O_2diff_ on study inclusion (i.e., transfused when the A-V O_2diff_ was > 3.7 mL and not transfused when the A-V O_2diff_ was ≤ 3.7 mL), had lower 90-day mortality, and organ function improved more rapidly in those in the “inappropriate” group. Moreover, RBC transfusions in patients with an A-V O_2diff_ ≤ 3.7 mL (“inappropriate” strategy) were associated with higher mortality, raising the hypothesis that transfusion may have harmful effects in patients without impaired oxygen reserve; this finding should be a starting point for future studies investigating whether avoiding RBC transfusion in patients without impaired oxygen reserve may not only be cost-saving but also clinically relevant [26].

Although some studies and many experts endorse the opinion that decisions to transfuse should not be based on Hb concentrations alone [7, 15, 16, 27–30], the latest guidelines based their recommendations only on this variable, suggesting that the Hb should be kept > 7 g/dL [10]. However, there is an individual response to low Hb concentrations, and patients with less physiological reserve are more vulnerable to anemia [15]. Differences in individual oxygen demand could partially explain the individual tolerance to anemia and, consequently, some of the conflicting results reported in the literature. A liberal transfusion strategy aimed at keeping Hb values > 9 g/dL was recently shown to be superior to a restrictive strategy in critically ill oncologic patients [31], and a recent clinical trial in vascular surgery suggested potential harm with a restrictive strategy, including lower cerebral tissue oxygenation and higher mortality rate [32]. However, a liberal strategy was associated with greater mortality in younger ICU patients (< 55 years) and those with an APACHE II score < 20 [28]. The A-V O_2diff_ may represent a good marker of a patient’s oxygen reserve, influenced by the patient’s comorbidities and oxygen demand. Indeed, we showed a reduction in mortality in the “appropriate” strategy group but no statistically significant difference in Hb level between the two groups; accordingly, within the same Hb range (7–10 g/dL), we observed great individual variability in the A-V O_2diff_.

The concept of a physiological transfusion trigger was introduced by Adamczyk et al., who proposed the use of ScvO_2_ as a tool to assess tolerance to anemia [33]. These authors showed that, in patients undergoing general anesthesia, RBC transfusion could result in a 5% increase in SvO_2_ only when pre-transfusion ScvO_2_ values were < 70%. We showed that basing the transfusion strategy on the difference between arterial and venous oxygen content, a physiological principle that reflects the balance between VO_2_ and DO_2_ can improve clinical outcomes. Additionally, we showed that the A-V O_2diff_ was a better prognostic factor than ScvO_2_, probably because the A-V O_2diff_ provides a more accurate estimation of the DO_2_ to VO_2_ ratio than does ScvO_2_ [34]. In support of this hypothesis, we also showed that O_2_ER, another surrogate of the DO_2_ to VO_2_ ratio, could be used to identify the appropriateness of a transfusion strategy, with an O_2_ER-based “inappropriate” strategy being independently associated with higher mortality. These results have some physiological background: the A-V O_2diff_ has been shown to be more accurate than ScvO_2_ in predicting tissue hypoxia [35]; further, the A-V O_2diff_ showed a strong inverse relationship with hemodilution [36], a well-known cause of unnecessary transfusion, whereas ScvO_2_ did not [37]. Finally, ScvO_2_ is considered a good estimator of oxygen extraction only when assuming that SaO_2_ = 1, but in hypoxemic patients, a low SaO_2_ can result in low ScvO_2_ values with normal A-V O_2diff_.

There was a trend towards more AKI with the “inappropriate” than with the “appropriate” strategy. Renal oxygen consumption is known to be high [38], so this organ may be particularly sensitive to an imbalance between DO_2_ and VO_2_. Moreover, RBCs can have beneficial effects on renal microvascular oxygenation [39]. Because the oxygen consumption of the kidney is so high, it is reasonable that an imbalance between DO_2_ and VO_2_ may affect this organ first. We also showed better organ function recovery in patients managed with an “appropriate” transfusion strategy, as shown by a more rapid decrease in SOFA score. RBC transfusions have a greater benefit in patients with severe organ failure, as reflected by a high SOFA score [7], but the average SOFA score at study inclusion did not differ in our two groups of patients. Therefore, in the presence of high A-V O_2diff_, RBC transfusion may be beneficial regardless of the baseline SOFA score. When transfused, patients with a high A-V O_2diff_ may also benefit from some relief of the heart when cardiac function is compromised [27]; of note, previous studies in animal models showed that treatments focused on improving myocardial VO_2_/efficiency were associated with improved ventricular function [40]. We did not explore cardiac function well enough to be able to evaluate this effect.

Our study has some limitations. First, the observational design does not enable us to establish a cause-effect relationship, and we cannot exclude the possibility that other clinical variables could have contributed to the difference in mortality described; a prospective randomized study would be needed for this purpose, and this study should therefore be considered as hypothesis generating. Second, because of the lack of published data in this field, we chose to separate patients into two groups based on median values, rather than trying to generate an optimal cutoff value; however, the Youden index analysis showed that the “best” A-V O_2diff_ cutoff identified, a value we could potentially have used to interpret the data, was very similar to the median value. Furthermore, the post hoc analysis performed using the “best” A-V O_2diff_ cutoff seemed to confirm our results. Third, the lack of hemodynamic monitoring did not enable us to separately investigate patients with and without preload dependency. We also did not systematically assess the clinical signs of cardiac congestion and thus cannot compare the occurrence of heart failure in the two groups. Future studies should address the reproducibility of our results in different hemodynamic settings. In this connection, we cannot exclude that a transfusion algorithm which considers clinical variables other than oxygen consumption (hemodynamic setting, lactate, SOFA score, and others) would improve our results; however, our study was not designed, and not powered enough, to develop such clinical tool. Similarly, we defined the occurrence of AKI according to the KDIGO guidelines, without including measurement of markers of kidney injury, such as cystatin C or neutrophil gelatinase-associated lipocalin; the addition of more specific markers may have strengthened our findings. Fourth, a recent meta-analysis showed that RBC transfusion can decrease O_2_ER in critically ill patients [41]. We did not systematically record A-V O_2diff_ and O_2_ER after transfusion and, therefore, cannot describe the A-V O_2diff_ and O_2_ER changes before and after RBC transfusion in the “appropriate” and “inappropriate” groups. In addition, although we showed a trend to an association of higher RDW values and mortality, as recently described [42], this was not confirmed in the multivariable analysis; nonetheless, there is a complex relationship between RDW values, iron deficiency, and RBC transfusion [43, 44], and our study was not designed to investigate this issue. Fifth, A-V O_2diff_ measurement requires a correctly placed central venous catheter; although widely used in critically ill patients, the central venous catheter is not always available or not placed in the superior vena cava. Finally, our transfusion rate (54%) was higher than that described in other studies [7], and we considered only patients who had a Hb concentration between 7.0 and 10.0 g/dL within 72 h after admission. External validation of our results and further analysis on patients with anemia later during the ICU stay are therefore needed.

## Conclusions

In anemic, non-bleeding critically ill patients, transfusion may be associated with lower 90-day mortality and morbidity in patients with higher A-V O_2diff_. These results support the hypothesis that transfusion therapy should be individualized according to the oxygen reserve of the patient.

## Supplementary information

**Additional file 1: Fig. S1.** Study definitions of “inappropriate” and “appropriate” transfusion strategies.

**Additional file 2: Fig. S2.** Flowchart of the study.

**Additional file 3; Fig. S3.** Daily changes in hemoglobin levels after study inclusion. Data are shown as mean ± standard deviation.

**Additional file 4: Fig. S4.** Daily assessment of SOFA score after study inclusion. + indicates *p*=0.048, * indicates *p*=0.002, # indicates *p*=0.001 comparing “inappropriate” with “appropriate” transfusion strategy

**Additional file 5: Table S1.** Clinical and demographic characteristics in survivors and non-survivors. **Table S2.** Univariate and multivariate analysis with 90-day mortality as dependent variable in non-transfused patients. **Table S3.** Secondary outcomes in the two groups. **Table S4.** Univariate and multivariate analysis with 90-day mortality as dependent variable. Appropriate group was defined using an oxygen extraction ratio (O_2_ER)-based strategy.

## Data Availability

The datasets generated during the current study are available from the corresponding author on reasonable request.

## Abbreviations

ICU: Intensive care unit
RBC: Red blood cell
VO_2_: Oxygen consumption
AKI: Acute kidney injury
TRALI: Transfusion-related acute lung injury
TACO: Transfusion-associated circulatory overload
DO_2_: Oxygen delivery
Hb: Hemoglobin
SvO_2_: Mixed venous oxygen saturation
A-V O_2diff_: Arterial-venous oxygen difference
SAPS: Simplified Acute Physiology Score
SOFA: Sequential Organ Failure Assessment
SAGM: Saline, adenine, glucose, mannitol
CaO_2_: Arterial oxygen content
CcvO_2_: Central venous oxygen content
O_2_ER: Oxygen extraction ratio

## References

[1] Khamiees M, Raju P, DeGirolamo A, Amoateng-Adjepong Y, Manthous CA (2001). Predictors of extubation outcome in patients who have successfully completed a spontaneous breathing trial. Chest.

[2] Malhotra R, Kashani KB, Macedo E, Kim J, Bouchard J, Wynn S, Li G, Ohno-Machado L, Mehta R (2017). A risk prediction score for acute kidney injury in the intensive care unit. Nephrol Dial Transplant.

[3] Rasmussen L, Christensen S, Lenler-Petersen P, Johnsen SP (2010). Anemia and 90-day mortality in COPD patients requiring invasive mechanical ventilation. Clin Epidemiol.

[4] Carson JL, Noveck H, Berlin JA, Gould SA (2002). Mortality and morbidity in patients with very low postoperative Hb levels who decline blood transfusion. Transfusion.

[5] Vlaar AP, Wolthuis EK, Hofstra JJ, Roelofs JJ, Boon L, Schultz MJ, Lutter R, Juffermans NP (2010). Mechanical ventilation aggravates transfusion-related acute lung injury induced by MHC-I class antibodies. Intensive Care Med.

[6] Bosboom JJ, Klanderman RB, Zijp M, Hollmann MW, Veelo DP, Binnekade JM, Geerts BF, Vlaar APJ (2018). Incidence, risk factors, and outcome of transfusion-associated circulatory overload in a mixed intensive care unit population: a nested case-control study. Transfusion.

[7] Vincent JL, Jaschinski U, Wittebole X, Lefrant JY, Jakob SM, Almekhlafi GA, Pellis T, Tripathy S, Rubatto Birri PN (2018). Worldwide audit of blood transfusion practice in critically ill patients. Crit Care.

[8] Hayden SJ, Albert TJ, Watkins TR, Swenson ER (2012). Anemia in critical illness: insights into etiology, consequences, and management. Am J Respir Crit Care Med.

[9] Van der Linden P, Rausin I, Deltell A, Bekrar Y, Gilbart E, Bakker J, Vincent JL (1995). Detection of tissue hypoxia by arteriovenous gradient for PCO2 and pH in anesthetized dogs during progressive hemorrhage. Anesth Analg.

[10] Mueller MM, Van RH, Meybohm P, Aranko K, Aubron C, Burger R, Carson JL, Cichutek K, De BE (2019). Patient blood management: recommendations from the 2018 Frankfurt Consensus Conference. JAMA.

[11] Carson JL, Terrin ML, Noveck H, Sanders DW, Chaitman BR, Rhoads GG, Nemo G, Dragert K, Beaupre L (2011). Liberal or restrictive transfusion in highrisk patients after hip surgery. N Engl J Med.

[12] Hebert PC, Wells G, Blajchman MA, Marshall J, Martin C, Pagliarello G, Tweeddale M, Schweitzer I, Yetisir E (1999). Transfusion Requirements in Critical Care Investigators, Canadian Critical Care Trials Group. A multicenter, randomized, controlled clinical trial of transfusion requirements in critical care. N Engl J Med.

[13] Villanueva C, Colomo A, Bosch A, Concepcion M, Hernandez-Gea V, Aracil C, Graupera I, Poca M, Alvarez-Urturi C (2013). Transfusion strategies for acute upper gastrointestinal bleeding. N Engl J Med.

[14] Murphy GJ, Pike K, Rogers CA, Wordsworth S, Stokes EA, Angelini GD, Reeves BC (2015). Liberal or restrictive transfusion after cardiac surgery. N Engl J Med.

[15] Vincent JL (2012). Indications for blood transfusions: too complex to base on a single number?. Ann Intern Med.

[16] Sakr Y, Vincent JL (2015). Should red cell transfusion be individualized?. Yes Intensive Care Med.

[17] Du Pont-Thibodeau G, Harrington K, Lacroix J (2014). Anemia and red blood cell transfusion in critically ill cardiac patients. Ann Intensive Care.

[18] Vallet B, Robin E, Lebuffe G (2010). Venous oxygen saturation as a physiologic transfusion trigger. Crit Care.

[19] von Elm E, Altman DG, Egger M, Pocock SJ, Gotzsche PC, Vandenbroucke JP (2008). The Strengthening the Reporting of Observational Studies in Epidemiology (STROBE) statement: guidelines for reporting observational studies. J Clin Epidemiol.

[20] Kozek-Langenecker SA, Afshari A, Albaladejo P, Santullano CA, De RE, Filipescu DC, Fries D, Gorlinger K, Haas T (2013). Management of severe perioperative bleeding: guidelines from the European Society of Anaesthesiology. Eur J Anaesthesiol.

[21] Yang WW, Thakkar RN, Gehrie EA, Chen W, Frank SM (2017). Single-unit transfusions and hemoglobin trigger: relative impact on red cell utilization. Transfusion.

[22] Spadaro S, Taccone FS, Fogagnolo A, Fontana V, Ragazzi R, Verri M, Valpiani G, Greco P, Bianconi M (2017). The effects of storage of red blood cells on the development of postoperative infections after noncardiac surgery. Transfusion.

[23] Cullis JO (2011). Diagnosis and management of anaemia of chronic disease: current status. Br J Haematol.

[24] Okusa MD, Davenport A (2014). Reading between the (guide)lines--the KDIGO practice guideline on acute kidney injury in the individual patient. Kidney Int.

[25] Fogagnolo A, Spadaro S, Creteur J, Cavalcante E, Taccone FS, Volta CA (2017). Can arterio-venous-oxygen content difference be a target to guide transfusion in critically ill patients?. Intensive Care Med Exp.

[26] Carson JL, Guyatt G, Heddle NM, Grossman BJ, Cohn CS, Fung MK, Gernsheimer T, Holcomb JB, Kaplan LJ (2016). Clinical practice guidelines from the AABB: red blood cell transfusion thresholds and storage. JAMA.

[27] Vincent JL, Van der Linden P (2016). Restrictive versus more liberal blood transfusions? The answer is in your heart. Minerva Anestesiol.

[28] Deans KJ, Minneci PC, Suffredini AF, Danner RL, Hoffman WD, Ciu X, Klein HG, Schechter AN, Banks SM (2007). Randomization in clinical trials of titrated therapies: unintended consequences of using fixed treatment protocols. Crit Care Med.

[29] Lelubre C, Vincent JL, Taccone FS (2016). Red blood cell transfusion strategies in critically ill patients: lessons from recent randomized clinical studies. Minerva Anestesiol.

[30] Chandra S, Kulkarni H, Westphal M (2017). The bloody mess of red blood cell transfusion. Crit Care.

[31] Bergamin FS, Almeida JP, Landoni G, Galas FRBG, Fukushima JT, Fominskiy E, Park CHL, Osawa EA, Diz MPE (2017). Liberal versus restrictive transfusion strategy in critically ill oncologic patients: the transfusion requirements in critically ill oncologic patients randomized controlled trial. Crit Care Med.

[32] Moller A, Nielsen HB, Wetterslev J, Pedersen OB, Hellemann D, Winkel P, Marcussen KV, Ramsing BGU, Mortensen A (2019). Low vs high hemoglobin trigger for transfusion in vascular surgery: a randomized clinical feasibility trial. Blood.

[33] Adamczyk S, Robin E, Barreau O, Fleyfel M, Tavernier B, Lebuffe G, Vallet B (2009). Contribution of central venous oxygen saturation in postoperative blood transfusion decision. Ann Fr Anesth Reanim.

[34] Squara P (2014). Central venous oxygenation: when physiology explains apparent discrepancies. Crit Care.

[35] Mekontso-Dessap A, Castelain V, Anguel N, Bahloul M, Schauvliege F, Richard C, Teboul JL (2002). Combination of venoarterial PCO2 difference with arteriovenous O2 content difference to detect anaerobic metabolism in patients. Intensive Care Med.

[36] Dubin A, Ferrara G, Kanoore Edul VS, Martins E, Canales HS, Canullan C, Murias G, Pozo MO, Estenssoro E (2017). Venoarterial PCO2-to-arteriovenous oxygen content difference ratio is a poor surrogate for anaerobic metabolism in hemodilution: an experimental study. Ann Intensive Care.

[37] Krantz T, Warberg J, Secher NH (2005). Venous oxygen saturation during normovolaemic haemodilution in the pig. Acta Anaesthesiol Scand.

[38] Ricksten SE, Bragadottir G, Redfors B (2013). Renal oxygenation in clinical acute kidney injury. Crit Care.

[39] Zafrani L, Ergin B, Kapucu A, Ince C (2016). Blood transfusion improves renal oxygenation and renal function in sepsis-induced acute kidney injury in rats. Crit Care.

[40] Santos-Gallego CG, Requena-Ibanez JA, San Antonio R, Ishikawa K, Watanabe S, Picatoste B, Flores E, Garcia-Ropero A (2019). Empagliflozin ameliorates adverse left ventricular remodeling in nondiabetic heart failure by enhancing myocardial energetics. J Am Coll Cardiol.

[41] Cavalcante Dos Santos E, Orbegozo D, Mongkolpun W, Galfo V, Nan W, Gouvêa Bogossian E, Taccone FS, Vallet B, Creteur J, Vincent JL (2019). Systematic review and meta-analysis of effects of transfusion on hemodynamic and oxygenation variables. Crit Care Med.

[42] Fogagnolo A, Spadaro S, Taccone FS, Ragazzi R, Romanello A, Fanni A, Marangoni E, Franchi F, Scolletta S, Volta CA (2019). The prognostic role of red blood cell distribution width in transfused and non-transfused critically ill patients. Minerva Anestesiol.

[43] Lasocki S, Lefebvre T, Mayeur C, Puy H, Mebazaa A, Gayat E, FROG-ICU study group (2018). Iron deficiency diagnosed using hepcidin on critical care discharge is an independent risk factor for death and poor quality of life at one year: an observational prospective study on 1161 patients. Crit Care.

[44] Spadaro S, Taccone FS, Fogagnolo A, Franchi F, Scolletta S, Ragazzi R, Fanni A, Marangoni E, Govoni M (2018). The effects of blood transfusion on red blood cell distribution width in critically ill patients: a pilot study. Transfusion.

