# Correction: Using arterial-venous oxygen difference to guide red blood cell transfusion strategy

**DOI:** 10.1186/s13054-022-04117-7

**Published:** 2022-08-24

**Authors:** Alberto Fogagnolo, Fabio Silvio Taccone, Jean Louis Vincent, Giulia Benetto, Elaine Cavalcante, Elisabetta Marangoni, Riccardo Ragazzi, Jacques Creteur, Carlo Alberto Volta, Savino Spadaro

**Affiliations:** 1 Department of Morphology, Surgery and Experimental Medicine, Section of Anaesthesia and Intensive Care, Azienda Ospedaliera-Universitaria Sant’ Anna, University of Ferrara, 8, Aldo Moro, 44121 Ferrara, Italy; 2 Department of Intensive Care, Erasme Hospital, Université Libre de Bruxelles, Brussels, Belgium

## Correction to: Critical Care (2020) 24:160 https://doi.org/10.1186/s13054-020-2827-5

In the publication of this article [1], in the Methods Section, there was an error in the following formulae:

$${\text{CaO}}_{2} = {\text{SaO}}_{2} \times {\text{Hb}} \times 1.39 + \left( {{\text{PaO}}_{2} \times 0.031} \right)$$

and

$${\text{CcvO}}_{2} = {\text{ScvO}}_{2} \times {\text{Hb}} \times 1.39 + \left( {{\text{PcvO}}_{2} \times 0.031} \right).$$

The correct formulae are:

$${\text{CaO}}_{2} = {\text{SaO}}_{2} \times {\text{Hb}} \times 1.39 + \left( {{\text{PaO}}_{2} \times 0.0031} \right)$$

and

$${\text{CcvO}}_{2} = {\text{ScvO}}_{2} \times {\text{Hb}} \times 1.39 + \left( {{\text{PcvO}}_{2} \times 0.0031} \right).$$

These have now been updated in the original article.

The data calculation used the correct formula.

We regret for any inconvenience that this inaccuracy may have caused.
