# Supplementary figures and images for: Using arterial-venous oxygen difference to guide red blood cell transfusion strategy
Source: Crit Care. 2020 Apr 20;24:160. doi: 10.1186/s13054-020-2827-5 (PMC7171832; doi:10.1186/s13054-020-2827-5)

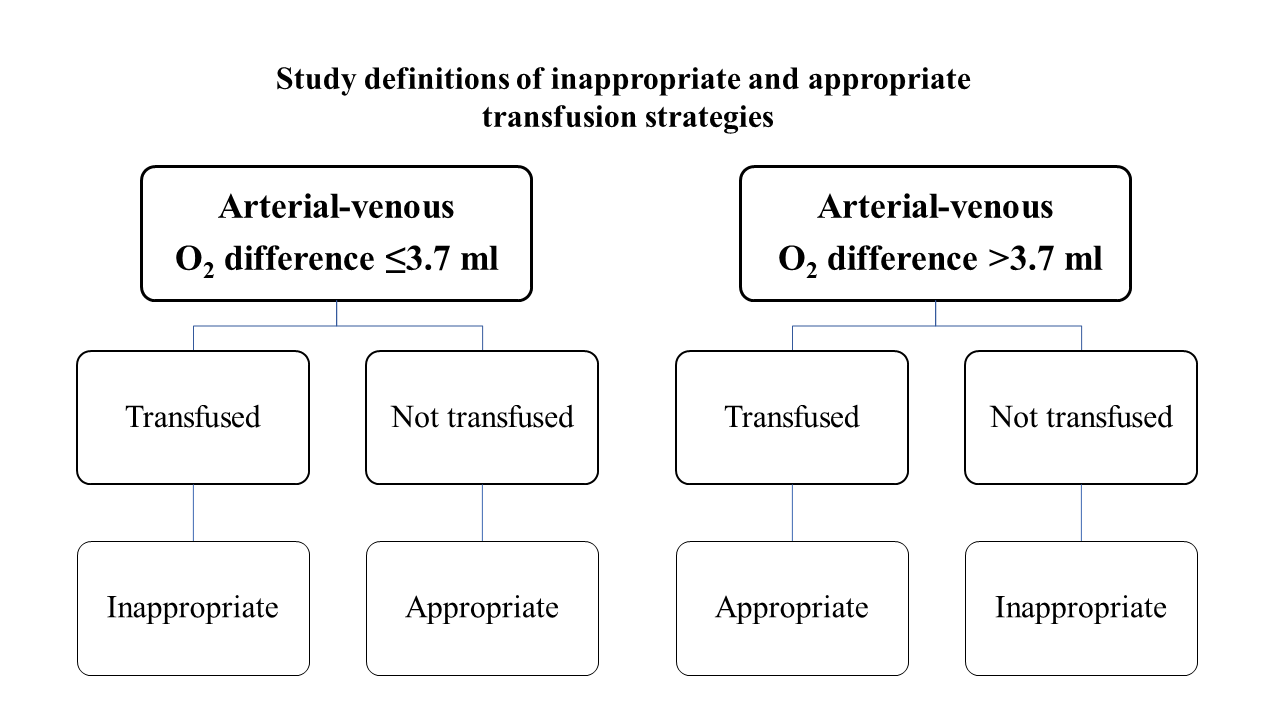

Supplement: Supplementary file 1 — Additional file 1: Fig. S1. Study definitions of “inappropriate” and “appropriate” transfusion strategies. [file 13054_2020_2827_MOESM1_ESM.tif]

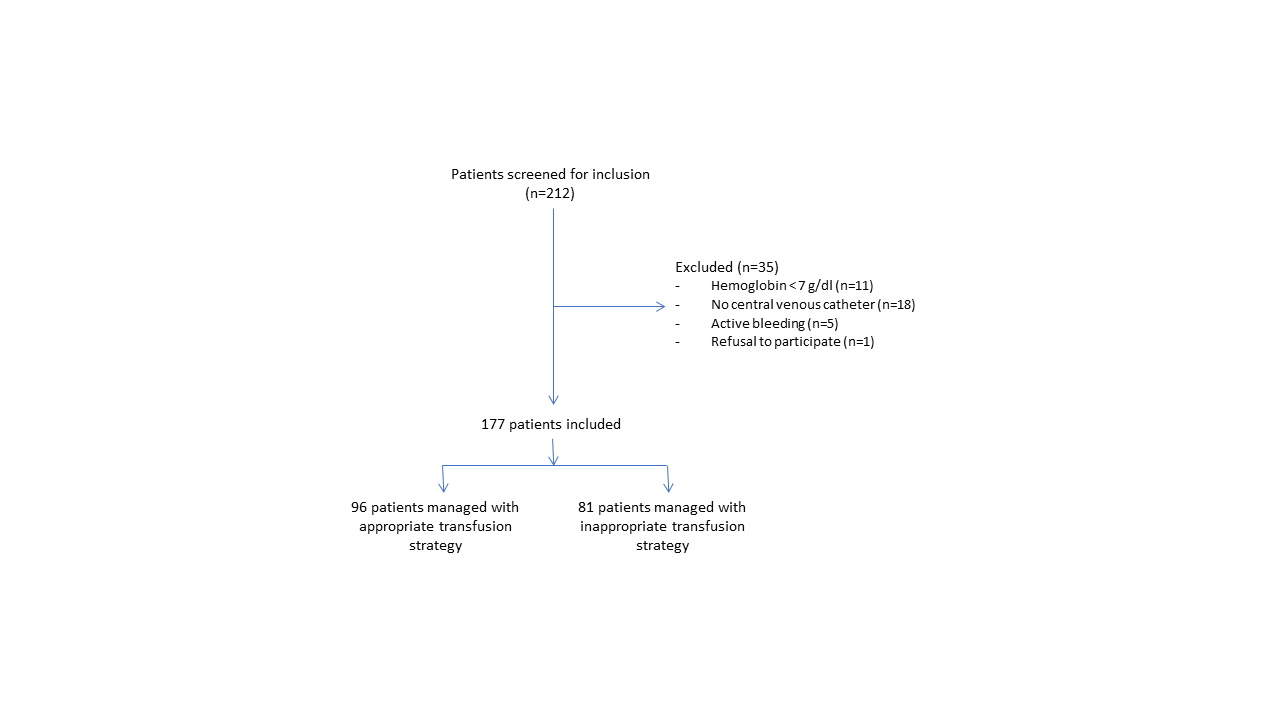

Supplement: Supplementary file 2 — Additional file 2: Fig. S2. Flowchart of the study. [file 13054_2020_2827_MOESM2_ESM.tif]
