# Supplementary material for: Using arterial-venous oxygen difference to guide red blood cell transfusion strategy
Source: Crit Care. 2020 Apr 20;24:160. doi: 10.1186/s13054-020-2827-5 (PMC7171832; doi:10.1186/s13054-020-2827-5)
Supplement: Supplementary file 5 — Additional file 5: Table S1. Clinical and demographic characteristics in survivors and non-survivors. Table S2. Univariate and multivariate analysis with 90-day mortality as dependent variable in non-transfused patients. Table S3. Secondary outcomes in the two groups. Table S4. Univariate and multivariate analysis with 90-day mortality as dependent variable. Appropriate group was defined using an oxygen extraction ratio (O2ER)-based strategy. [file 13054_2020_2827_MOESM5_ESM.docx]

**Using arterial-venous oxygen difference to guide red blood cell transfusion strategy**

Fogagnolo et al

Online Supplement

**Supplemental Table 1.** Clinical and demographic characteristics in survivors and non-survivors

| **Characteristic** | **Survivors**  **(n=118)** | **Non-survivors**  **(n=59)** | **p** |
| --- | --- | --- | --- |
| Age, years | 70 ± 13 | 74 ± 10 | 0.09 |
| BMI, kg/m^2^ | 28 ± 5 | 31 ± 5 | 0.17 |
| SAPS II | 40.8 ± 14.4 | 45.5 ± 11.1 | 0.06 |
| Transfused, n (%) | 64 (54) | 31 (52) | 0.87 |
|  |  |  |  |
| *Comorbidity* |  |  |  |
| Heart disease, n (%) | 70 (59) | 32 (54) | 0.58 |
| Hypertension, n (%) | 77 (66) | 41 (69) | 0.75 |
| Diabetes, n (%) | 31 (26) | 22 (37) | 0.19 |
| COPD/asthma, n (%) | 24 (20) | 6 (10) | 0.13 |
| History of smoking, n (%) | 33 (28) | 12 (20) | 0.34 |
| Chronic renal disease, n (%) | 31 (26) | 22 (37) | 0.19 |
|  |  |  |  |
| *Interventions on admission* |  |  |  |
| Mechanical ventilation, n (%) | 101 (86) | 56 (94) | 0.11 |
| Vasopressors, n (%) | 42 (35) | 39 (66) | <0.001 |
|  |  |  |  |
| *Laboratory values on inclusion* |  |  |  |
| Hemoglobin, g/dL | 8.7 ± 0.7 | 8.8 ± 0.7 | 0.42 |
| A-V O_2_ _diff_, mL | 3.7 [2.6 – 4.4] | 3.6 [2.4 – 4.2] | 0.64 |
| Oxygen extraction ratio, % | 28 ± 9 | 28 ± 12 | 0.74 |
| MCV, fL | 89 ± 9 | 87 ± 8 | 0.22 |
| RDW, % | 15.5 ± 2.4 | 16.7 ± 3.7 | 0.01 |
| Platelets, 10^3^ µl^-1^ | 192 [124 – 280] | 158 [136 – 222] | 0.42 |
| INR | 1.2 ± 0.3 | 1.3 ± 0.3 | 0.61 |
| Creatinine, mg/dL | 1.08 [0.90 – 2.00] | 1.09 [0.78 – 2.51] | 0.84 |
| Bilirubin, mg/dL | 0.69 [0.34 – 1.10] | 0.76 [0.50 – 1.10] | 0.17 |
| Lactate, mmol/L | 1.4 [1.1 – 2.0] | 1.6 [1.1 – 3.0] | 0.66 |
| PaO_2_/F_I_O_2_ ratio | 278 [219 – 367] | 261 [162 – 380] | 0.90 |

BMI: body mass index; SAPS: Simplified Acute Physiology Score; COPD: chronic obstructive pulmonary disease; RDW: red blood cell distribution width; MCV: mean corpuscular volume; INR: international normalized ratio. CRP: C-reactive protein; PCT: procalcitonin; ScvO_2_: central venous oxygen saturation

**Supplemental Table 2.** Univariate and multivariate analysis with 90-day mortality as dependent variable in non-transfused patients.

| **Variables** | **Unadjusted**  **Odds ratio** | **p value** | **Adjusted**  **Odds ratio** | **p value** |
| --- | --- | --- | --- | --- |
| A-V O_2diff_ , mL | 1.50 [1.02 – 2.20] | 0.02 | 1.52 [1.00– 2.35] | 0.05 |
| SOFA score | 1.50 [1.19 – 1.91] | 0.001 | 1.51 [1.18 – 1.93] | 0.001 |
| SAPS II | 1.03 [0.99 – 1.07] | 0.06 | 1.03 [0.99 – 1.07] | 0.14 |

SAPS: Simplified Acute Physiology Score;

**Supplemental Table 3.** Secondary outcomes in the two groups

| **Variables** | **“Appropriate” Strategy**  **(n=96)** | **“Inappropriate” Strategy**  **(n=81)** | **p value** |
| --- | --- | --- | --- |
| Length of stay in ICU, days | 8 [5 – 16] | 7 [4 – 12] | 0.23 |
| Length of stay in hospital, days | 18 [12 – 24] | 17 [12 – 24] | 0.78 |
| Number of RBC during the first 5 days | 1 [1 – 1] | 1 [0 – 1] | 0.46 |
| Number of patients with at least 1 RBC during the first 5 days, n (%) | 70 (73%) | 55 (68%) | 0.53 |
| Acute kidney injury, n (%) | 13/96 (13%) | 21/81 (26%) | 0.06 |
| Mechanical ventilation free days | 4 [1 – 10] | 3 [1 – 10] | 0.49 |
| Vasopressor free days | 0 [0 – 2] | 1 [0 – 4] | 0.23 |

ICU: Intensive care unit.

**Supplemental Table 4.** Univariate and multivariate analysis with 90-day mortality as dependent variable. Appropriate group was defined using an oxygen extraction ratio (O_2_ER)-based strategy.

| **Variables** | **Unadjusted**  **Odds ratio** | **p value** | **Adjusted**  **Odds ratio** | **p value** |
| --- | --- | --- | --- | --- |
| SAPS II | 1.03 [1.00 – 1.06] | 0.02 | 1.01 [0.99 – 1.04] | 0.18 |
| O_2_ER- based appropriate group | 0.40 [0.21 – 0.77] | 0.006 | 0.44 [0.23 – 0.86] | 0.02 |
| Lactate, mmol/L | 1.40 [1.07 – 1.82] | 0.01 | 1.17 [0.92 – 1.51] | 0.20 |

BMI: Body Mass Index; SAPS: Simplified Acute Physiology Score; COPD: Chronic obstructive pulmonary disease; RDW: Red blood cell distribution width; INR: International normalized ratio; PaO_2_: Partial pressure of oxygen; F_I_O_2_: Fraction of inspired oxygen

**Supplemental sub-group analyses**

1.

Patients with hemoglobin ≤8.5 (n=72)

Univariate analysis with 90-day mortality as dependent variable. Appropriate transfusion strategy OR=0.34 [95% CI 0.12 – 0.97] p=0.04

Patients with hemoglobin >8.5 (n=105)

Univariate analysis with 90-day mortality as dependent variable. Appropriate transfusion strategy OR=0.43 [95% CI 0.18 – 0.98], p=0.04

2

Univariate analysis in transfused patients with 90-day mortality as dependent variable and A-V O_2diff_ as continuous variable. OR=0.73 [95% CI 0.52 – 1.02] p=0.08
